# A deep learning system for prostate cancer diagnosis and grading in whole slide images of core needle biopsies

**DOI:** 10.1038/s41598-022-07217-0

**Published:** 2022-03-01

**Authors:** Nitin Singhal, Shailesh Soni, Saikiran Bonthu, Nilanjan Chattopadhyay, Pranab Samanta, Uttara Joshi, Amit Jojera, Taher Chharchhodawala, Ankur Agarwal, Mahesh Desai, Arvind Ganpule

**Affiliations:** 1 AIRAMATRIX PVT. LTD., Mumbai, India; 2 Muljibhai Patel Urological Hospital, Nadiad, India; 3 Grant Medical Foundation, Ruby Hall Clinic, Pune, India; 4 Department of Lab Medicine, Manipal Hospital, Jaipur, India

**Keywords:** Cancer, Urological cancer, Prostate cancer, Oncology, Cancer, Urological cancer, Prostate cancer, Engineering, Biomedical engineering

## Abstract

Gleason grading, a risk stratification method for prostate cancer, is subjective and dependent on experience and expertise of the reporting pathologist. Deep Learning (DL) systems have shown promise in enhancing the objectivity and efficiency of Gleason grading. However, DL networks exhibit domain shift and reduced performance on Whole Slide Images (WSI) from a source other than training data. We propose a DL approach for segmenting and grading epithelial tissue using a novel training methodology that learns domain agnostic features. In this retrospective study, we analyzed WSI from three cohorts of prostate cancer patients. 3741 core needle biopsies (CNBs) received from two centers were used for training. The κquad (quadratic-weighted kappa) and AUC were measured for grade group comparison and core-level detection accuracy, respectively. Accuracy of 89.4% and κquad of 0.92 on the internal test set of 425 CNB WSI and accuracy of 85.3% and κquad of 0.96 on an external set of 1201 images, was observed. The system showed an accuracy of 83.1% and κquad of 0.93 on 1303 WSI from the third institution (blind evaluation). Our DL system, used as an assistive tool for CNB review, can potentially improve the consistency and accuracy of grading, resulting in better patient outcomes.

## Introduction

Prostate cancer is the most commonly diagnosed cancer in men and one of the leading causes of death from cancer^1^. Despite its prevalence, prostate cancer is often non-aggressive, making it difficult to establish whether the disease offers a high enough risk to patients to warrant treatments like prostate surgery (prostatectomy) or radiation therapy. Histopathologically assigned Gleason grades are one of the most powerful prognostic predictors in prostate carcinoma. However, Gleason grading is difficult to perform and subjective with significant inter-and intra-observer variability^2^. While, uropathologists have a higher rate of agreement^3^, this skill is not frequently available. Recent recommendations require pathologists to estimate and quantify the percentage of tumor across multiple Gleason patterns, add to the burden for pathologists and exacerbate the subjectivity issues^4^.

The Gleason scoring system divides prostate cancer into risk groups based on the Gleason score, ranging from 3 + 3 (low risk) to 5 + 5 (high risk). On a Core Needle Biopsy (CNB), the Gleason score is the sum of the most common primary Gleason pattern and the highest secondary pattern, as determined by the pathologist on histopathological examination. The notion of five prognostically distinct grade groups was established in the most recent iteration of the Gleason grading system^5^, and assigns scores 3 + 3 and lower to group 1; 3 + 4 to group 2; 4 + 3 to group 3, 3 + 5, 5 + 3 and 4 + 4 to group 4; and the remaining higher grades to group 5. Although the new system includes fewer groups, research shows that the inter-and intra-observer variability has not decreased^2^.

Research has shown that computational pathology and deep learning techniques can perform expert-level pathology diagnosis^6,7^. Several studies have shown that deep learning-based algorithms can handle Gleason scoring with performance comparable to expert-provided diagnosis^8–16^. All of these techniques describe accuracy and agreement solely in terms of core-level grading, ignoring gland-level segmentation and the overlap with pathologists’ pixel-level annotations. Furthermore, most of these methods are limited in their applicability to data distributions that haven't been seen during training.

**Figure 1:**
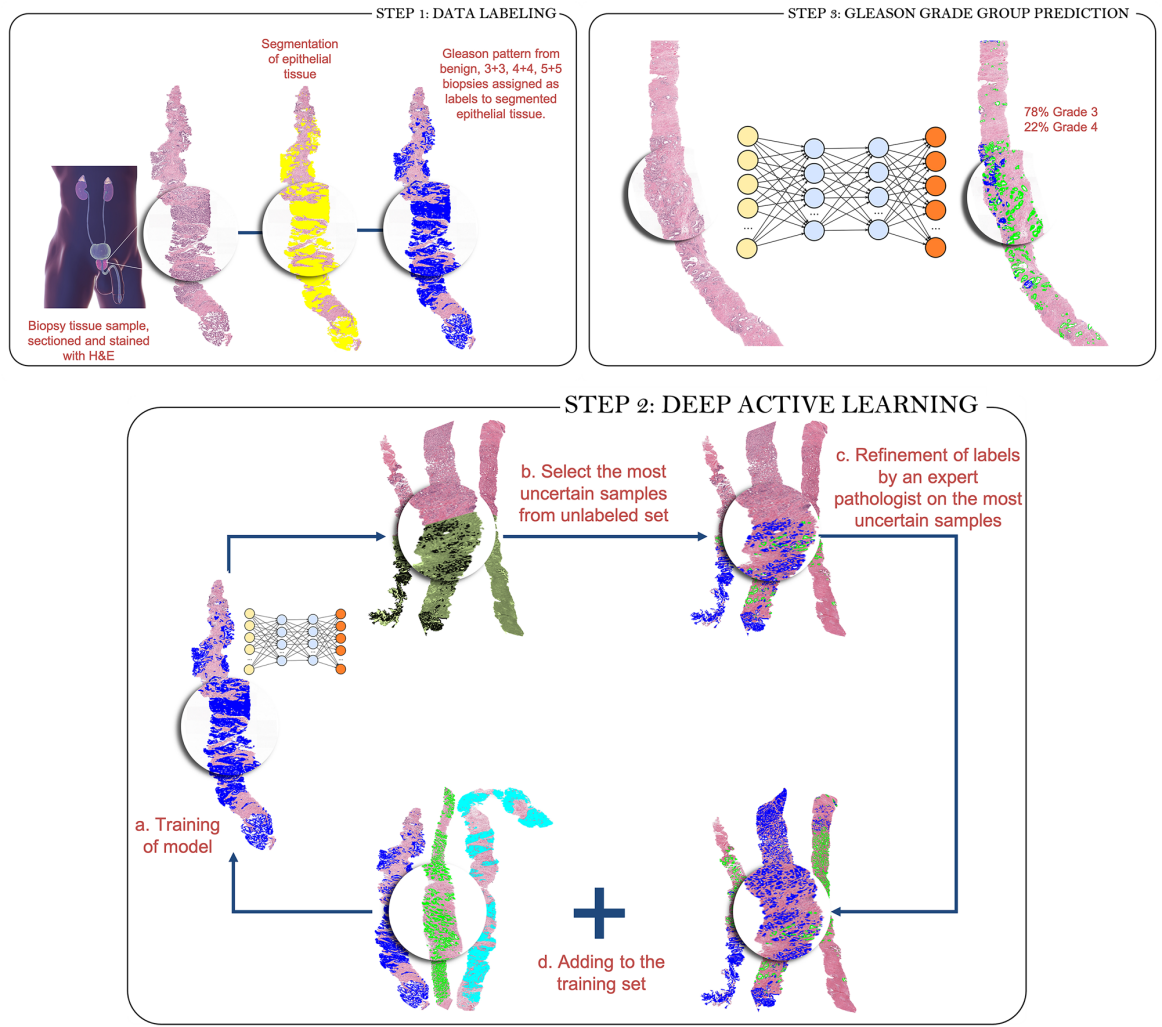
An overview of the deep learning system's development. To produce pixel-level annotations, we adopt a semi-supervised technique that employs Active Learning. The final system assigns a Gleason grade group at the core level and also shows pixel-level overlays. These overlays can be used as a second read by pathologists to ensure that no image areas are overlooked during processing.

Our work presents a Deep Learning system (Fig. 1) to detect cancer regions and predict Gleason grades in WSI of prostate CNB. Our system is a semi-supervised method that uses Active Learning and uncertainty measures to select samples for annotation. We also describe a Convolutional Neural Network (CNN) architecture that learns domain-agnostic features for greater generalization. We evaluate our approach on a large cohort of patients with a consensus reference standard. To create a consensus reference standard, we requested a panel of pathologists to annotate an internal test set. Using Cohen's quadratic kappa metric, we compared results of our technique to the consensus reference. In addition, we compared our approach to two external test sets released by RUMC and Karolinska^17^. The test set from Karolinska was not used during training to assess the generalizability of our system. Finally, the system was tested for its capacity to distinguish between benign and malignant biopsies, biopsies with low- and high-grade tumors, and group 2 vs. group 3 biopsies.

## Data

A total of 6670 WSI were used in this investigation, sourced from Muljibhai Patel Urological Hospital, Radboud University Medical Center, and Karolinska Institute. Cases were randomly assigned to either the development (training/tuning) or independent validation datasets.

### Muljibhai Patel Urological Hospital (MPUH) data set

The pathology archives of the MPUH provided de-identified, anonymized 580 CNB slides from 110 individuals. Hematoxylin and Eosin (H&E)-stained formalin-fixed paraffin-embedded (FFPE) needle core biopsies were de-identified, and digital whole-slide images were obtained. The MPUH Institutional Review Board (IRB), # EC/678/2020, authorized the study protocol and waived informed consent because the data was de-identified and utilized for a retrospective study without affecting patient care. A Hamamatsu Nanozoomer XR was used to digitize CNB slides at 40 × magnification. The dataset was split into training (155) and testing sets (425). The ISUP grades group distribution in the MPUH dataset is shown in Appendix 2a of the supplementary material.

### Prostate cANcer graDe assessment (PANDA) challenge dataset

PANDA is a publicly available dataset^17^ created as part of a Kaggle competition held by Radboud University Medical Center and Karolinska Institute in collaboration with Tampere University. We used 3586 biopsies from Radboud University Medical Center for training and 1201 for testing. In addition, 1303 biopsies from Karolinska Institute were used as unseen test data.

## Results

### Inter-observer agreement

A panel of pathologists evaluated the internal set of 425 biopsies to determine the Gleason grade. These biopsies were graded separately by four pathologists (two uropathologists and two general surgical pathologists). The inter-observer agreement between the two uropathologists was 0.89 (κ_quad_). The agreement between general surgical pathologists was 0.69 (κ_quad_). The agreement between uropathologists and general surgical pathologists was in the range of 0.50 ~ 0.59 (κ_quad_). On the Gleason grade group, the mean inter-observer agreement was 0.79 (κ_quad_) with the consensus reference from a panel of four pathologists. The individual pathologists' scores are listed in Table 1.

**Table 1:** Agreement with the consensus reference standard.

Metric	Uropathologist-1	Uropathologist-2	General surgical pathologist-1	General surgical pathologist-2
Quadratic kappa	0.9014	0.9112	0.7112	0.6456

### Performance analysis

**Figure 2:**
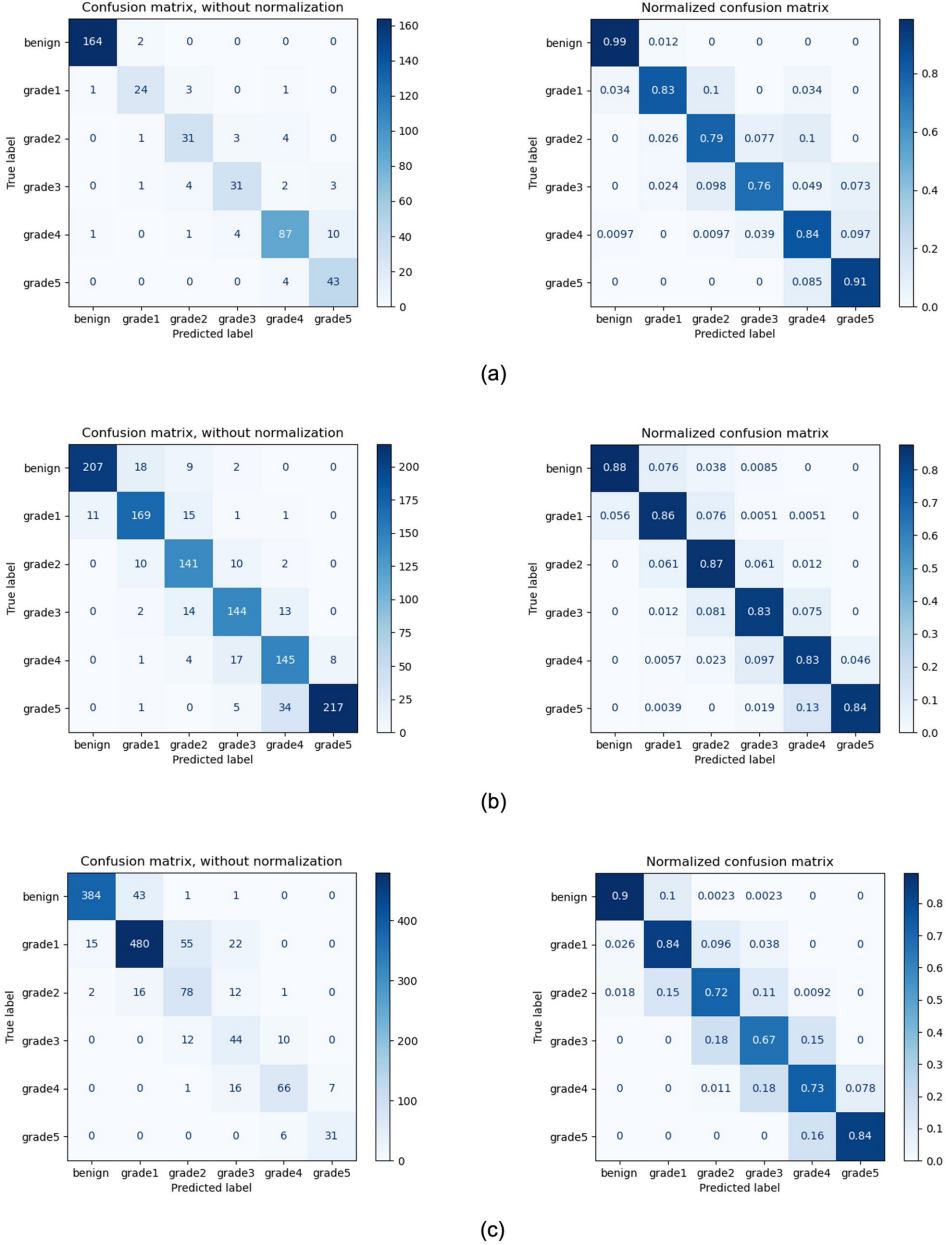
Gleason grade group confusion matrices for test sets (**a**) MPUH; (**b**) Radboud; (**c**) Karolinska. For each set, the quadratic Cohen's kappa is displayed.

Our proposed system demonstrated an accuracy of 89.4 percent and 0.92 agreement (κ_quad_) with the consensus on grading of the 425 biopsies in the test set. For the external test set from Radboud, the accuracy was 85.3 percent with a κ_quad_ of 0.96. On the external test set from Karolinska, where the system was not exposed to this data in training, the model demonstrated an accuracy of 83.1 percent and a κ_quad_ of 0.93. The confusion matrix between core-level annotations derived from different test sets and predictions made by the proposed system is shown in Fig. 2. The receiver operating characteristic (ROC) curves for detecting grade groups are shown in Appendix 2b of the supplementary material.

**Figure 3:**
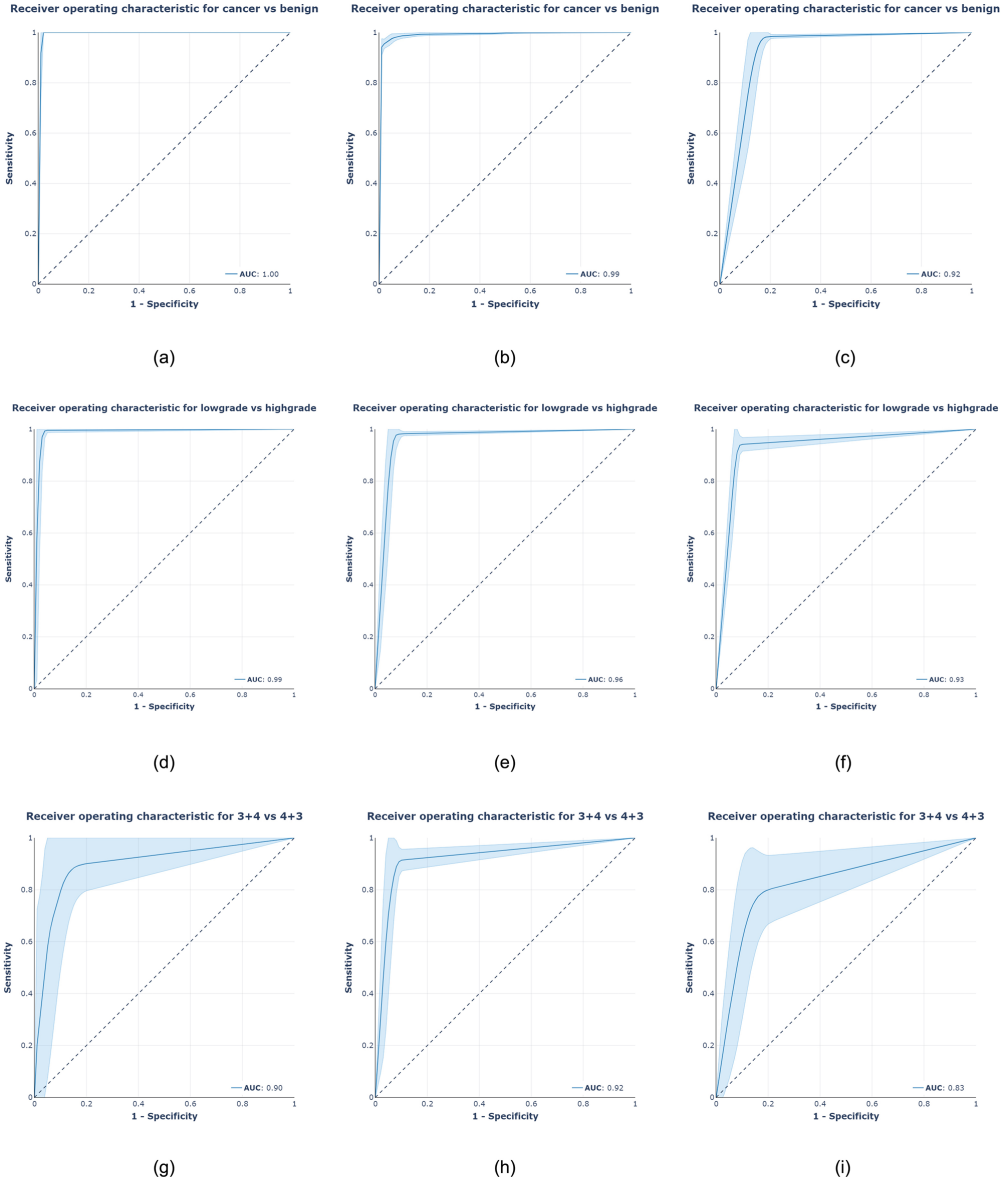
ROC analysis of clinically significant parameters: (first row) cancer vs. benign; (middle row) low-grade vs. high-grade tumors; (bottom row) grade group 2 vs. grade group 3. The results of the MPUH test set are displayed in the first column. The findings of the Radboud test set are shown in the middle column, and the results of the Karolinska set are shown in the last column, comparing our system's predictions to the pathologists' reference.

We also utilized ROC analysis to test if the system could classify images into clinically relevant categories such as benign vs. malignant, low-grade vs. high-grade, and group 2 vs. group 3 biopsies. The system attained AUC values of 0.997, 0.991, and 0.920 percent for test sets of MPUH, Radboud, and Karolinska respectively, in the core-level cancer detection task. In addition, the method demonstrated an AUC of 0.990, 0.960, and 0.930 percent for low-grade (group 2) vs. high-grade (grade group > 2) tumor biopsies on three test sets. The system achieved AUCs of 0.900, 0.920, and 0.830 on the three test sets respectively, when compared between group 2 (3 + 4) and group 3 (4 + 3). Figure 3 depicts the ROC curves for the three clinically relevant categories and three test sets.

## Methods

As shown in Fig. 1, the suggested method for automated Gleason grading comprises of three major steps. A pathologist selects and annotates a limited fraction of images with pathologist Gleason grades 3 + 3, 4 + 4, and 5 + 5 for tumor glands. To segment the epithelial tissue, a fully convolutional network (see Sect. “Fully convolutional neural network”) is trained. The pathologist's Gleason grade is assigned to the predicted tumor locations. In the following stage, these predictions are used as labels. In step 2, the system simulates iterative active learning-based data labelling on datasets. This simulation environment was inspired by the Cost-Effective Active Learning paradigm described in^18^. Using the initial labelled dataset, a Fully Convolutional Network (FCN) semantic segmentation model (the same design as the epithelium detection segmentation model) is trained for Gleason grade group identification in the first iteration. Following training, the system is capable of segmenting glands based on Gleason patterns 3, 4, and 5. Unlabeled images are subsequently fed into the trained FCN, and a measure of uncertainty is computed for each unlabeled sample. A pathologist then annotates the most uncertain samples (with uncertainty measure more than the threshold) and adds them to the initial training set. In the following iteration, the FCN is re-trained with a new set of annotated images. This step is repeated until no samples have an uncertainty measure greater than the threshold. Appendix 1d of the supplementary material present the pseudocode of the above deep active learning step. In the final step, the model from the last iteration is used to predict the Gleason pattern on the test set.

The subsections that follow discuss the fully convolution network used in this work, as well as the uncertainty measure computation and domain agnostic training methods used to reach generalised results. The MPUH Institutional Review Board (IRB), # EC/678/2020, authorized the study protocol, waived informed consent because the data was de-identified and utilized for a retrospective study without affecting patient care, and approved the experiments. All the experiments were performed in accordance with relevant guidelines and regulations of the IRB.

### Fully convolutional neural network

The network is built on a u-shaped architecture U-Net^19^ and comprises a contracting path and an expansive path. The contracting path is a conventional CNN consisting of convolutions applied repeatedly, followed by a rectified linear unit (ReLU) and a max-pooling operation. The expansive pathway combines feature and spatial information through a series of up-convolutions and concatenations using high-resolution features from the contracting path. ResNext50^20^ replaces vanilla U-Net's contracting path, commonly known as the encoder. It's a homogeneous neural network that uses "cardinality", an additional dimension on top of ResNet's^21^ breadth and depth, to reduce the number of hyperparameters required by traditional ResNet. In addition, ResNext design combines ResNet's repetition of residual block method with Inception Network's split-transform-merge strategy, resulting in better performance.

To increase performance even more, Atrous spatial pyramid pooling (ASPP)^22^ has been implemented into U-Net. The ASPP module is added after the Encoder–Decoder network's bottleneck, i.e. the encoder's feature map is processed using ASPP and the result is then supplied to the decoder. ASPP is a hybrid of atrous convolution and spatial pyramid pooling that can gather contextual data at various scales for more precise classification. It aids in processing the original image with various filters with complementing effective fields of view and capturing both objects and meaningful image context at multiple scales. A feature pyramid network (FPN) is included to aid in the segmentation of very small glands. The FPN blends low-resolution, semantically strong features with high-resolution, weak features using a top-down approach, and lateral connections.

Multiple models with this architecture are then trained on five folds of data with various stain-based augmentations. The aggregate knowledge of five base models is then transferred using ensemble distillation into a student network using the modified U-Net architecture discussed above.

### Uncertainty measure

A Bayesian counterpart of an FCN can be used to determine a model's confidence in a sample prediction. We adopted an approach known as Monte-Carlo dropout^23^, where we can sample from the approximate posterior by keeping the dropout active during prediction time and repeating forward passes. Because of the stochasticity of dropout, various neurons will be triggered or silenced, resulting in slightly different predictions. This helps the Bayesian FCN model approximate variational inference. The standard deviation of the N posterior probabilities is then used to generate the uncertainty map. Finally, the overall uncertainty measure for an unlabeled sample is calculated by adding the values of the pixels in the uncertainty maps.

### Domain agnostic training

The stain color distribution of a WSI is influenced by various factors like tissue slide preparation, fixation, processing, sectioning and staining procedures, and scanning equipment. As a result, there is a lot of variation in the appearance of histopathology images from laboratory to laboratory, as shown in Appendix 2c of the supplementary material. The performance of deep learning models on out-of-distribution samples is hampered due to this domain shift between images acquired from various laboratories^24^.

**Figure 4:**
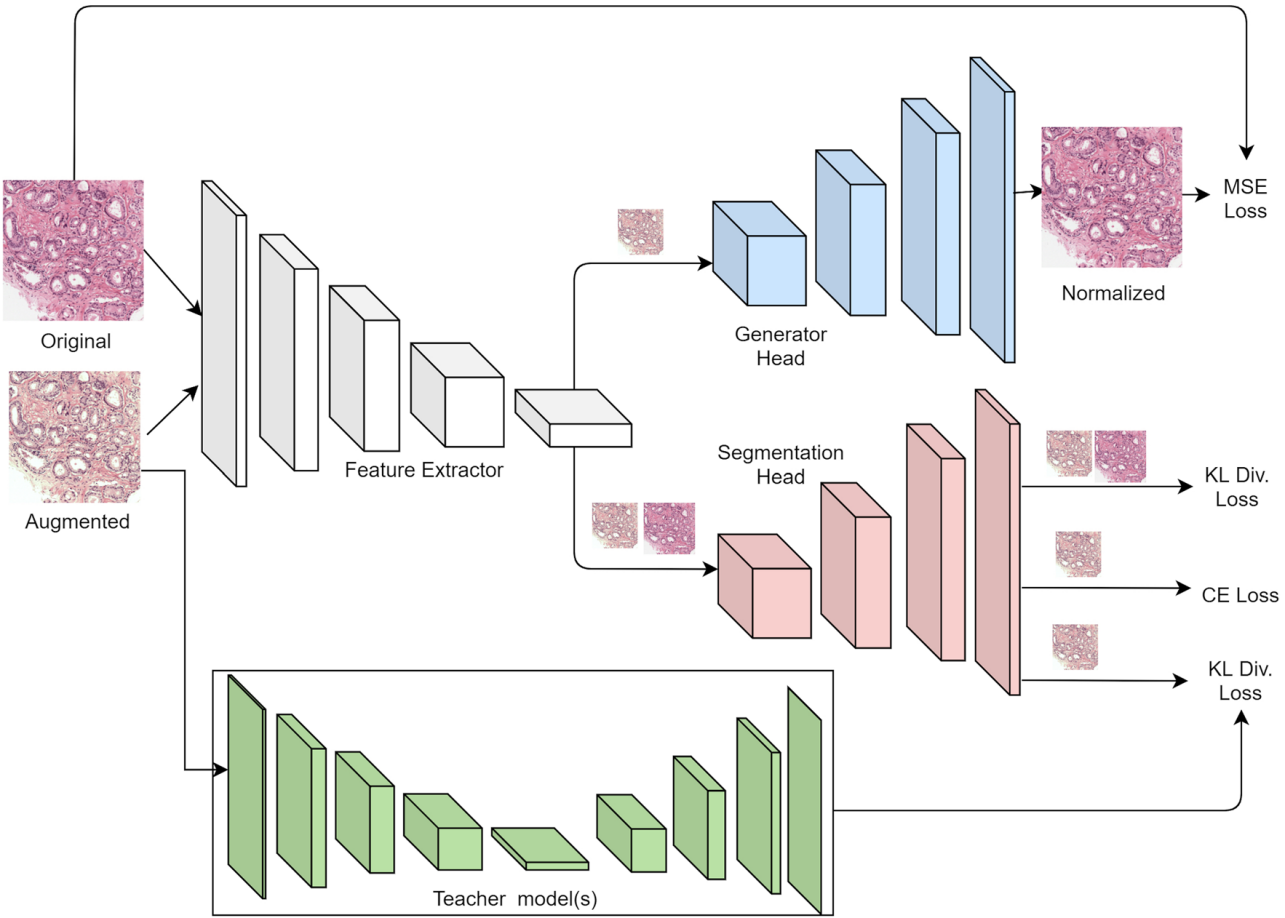
Proposed Multi-Task Model for learning features robust to color stain variations in histopathology images. During the training phase, the model learns from three supervisory signals—reconstruction loss between raw and color augmented image, KL divergence loss between raw and color augmented image logits, and segmentation DICE loss. At inference, only the layers required for segmentation are used.

We present a domain-neutral training methodology using a multi-task paradigm. As shown in Fig. 4, we train a segmentation model (FCN as described in Sect. “Fully convolutional neural network”) that shares its feature extractor with a stain-normalization network (Generator Head). We present the multi-task model with a pair of raw and color augmented images that simulate stain color variations during the training period. The model reconstructs a normalized image from the color augmented image that matches the raw image while also semantically segmenting the image into multiple pixel-level class labels. To enforce learning stain robust features, we penalize the distance between the logits for color augmented and raw images. As a result, the model is guided through stain normalization and accurately detecting the augmented image. To mitigate the effect of label noise, multiple models with the FCN architecture (Teacher Models) are trained on five folds of data with different stain-based augmentations. The aggregate knowledge of the five base models is then transferred into a student network via ensemble distillation utilising KL divergence loss between the logits of the colour augmented image in the student network and the average of the five Teacher models. This stage of ensemble distillation acts as a strong regularizer, reducing the effect of label noise in the training set. During training, the overall loss function is a weighted combination of the MSE loss between stain normalised and original image, the KL divergence loss between colour augmented and raw image, the Cross-entropy pixel segmentation loss, and the KL divergence ensemble distillation loss. The layers allocated to stain normalisation are deleted during prediction on the test set, leaving only the layers required for image segmentation.

### Algorithm performance analysis

Appendix 1a, 1b, 1c of the supplementary material discusses implementation specifics, color augmentation technique, and training parameters, respectively. The performance of the proposed ensemble distillation model against state-of-the-art FCN architectures is shown in Table 2. The proposed model outperforms U-Net++^25^, DeepLabV3+^26^, and U-Net-ASPP-FPN^27^ on both the MPUH and Radboud datasets. We report pixel-level accuracy, F1-score using stroma, benign, grade 3, grade 4, and grade 5 as the positive labels. The proposed method achieved an overall F1-score of 0.898 and 0.823 on the MPUH and Radboud sets, respectively. The Karolinska Institute dataset lacked pixel-level labels and was excluded from the segmentation performance evaluation. Based on this analysis, we can infer that ensemble knowledge distillation favourably boosts segmentation performance. When compared to the MPUH dataset, the Radboud dataset's F1-score exhibited a decline in performance. The cause of this performance drop could be attributed to label noise. We quantified the label noise in the Radboud dataset by having students annotate the images. The labels determined by the students were compared to the experts' consensus labels. In the grade group, the accuracy was 72% (κ_quad_ 0.85)^17^. These numbers reveal the existence of label errors. The MPUH dataset was annotated by a group of expert pathologists and had low label noise. The performance of core-level CNB classification against state-of-the-art FCN architectures is shown in Appendix 2d of the supplementary material. On all three data cohorts, the proposed approach outperforms state-of-the-art networks.

**Table 2:** Segmentation results on the (a) MPUH; (b) Radboud test sets.

(a)				
Classes	UNet + FPN + ASPP — ResNeXt50	Ours	UNet++ — EfficientNetB4	DeepLabV3+
Benign	0.852	0.86	0.887	0.887
Grade 3	0.881	**0.897**	0.836	0.856
Grade 4	0.90	**0.912**	0.905	0.889
Grade 5	0.923	0.923	0.95	0.952

(b)				
Classes	UNet + FPN + ASPP — ResNeXt50	Ours	UNet++ — EfficientNetB4	DeepLabV3+
Benign	0.721	0.745	0.673	0.759
Grade 3	0.849	0.830	0.712	0.726
Grade 4	0.802	0.795	0.584	0.787
Grade 5	0.920	**0.925**	0.676	0.767

### Qualitative results

Appendix 2e shows a few sample cases from the internal test set. The final system assigns a Gleason grade group at the core level and also shows pixel-level overlays. These overlays can be used as a second read by pathologists to ensure that no image areas are overlooked during processing. Appendix 2f depicts illustrative patches from system failure instances (false positive and negative). (a) shows a Cribriform grade 4 pattern classified as a grade 5 pattern; (b) shows a case of lymphocyte infiltration incorrectly predicted by the system as a grade 5 case; (c) shows a grade 4 case classified as a grade 5 case; (d) Cutting artefact predicted by the system as a false positive in this case. The area which had compressed, and dense tissue was apparent on the biopsy's margin.

## Discussion

We created a new Deep Learning system to detect cancer areas and predict Gleason grades on WSI of prostate CNBs. The suggested system employs a domain-agnostic training methodology in conjunction with a Deep Active Learning framework. Given the importance of pathology review in predicting patient outcome, and the scarcity of specialty pathologists, the use of Deep Learning systems as an assistive tool for prostate CNB review has the potential to improve access to consistent, accurate grading and lead to better patient outcomes. Our system's exact gland-level segmentation allows us to calculate Gleason grade area percentages and define grade groups. Further, pathologists can utilize pixel-level overlays as a second read to ensure that no image areas are missed from the analysis. Our strategy is similar to that employed by pathologists in clinical practice and significantly improves the system's interpretability.

**Figure 5:**
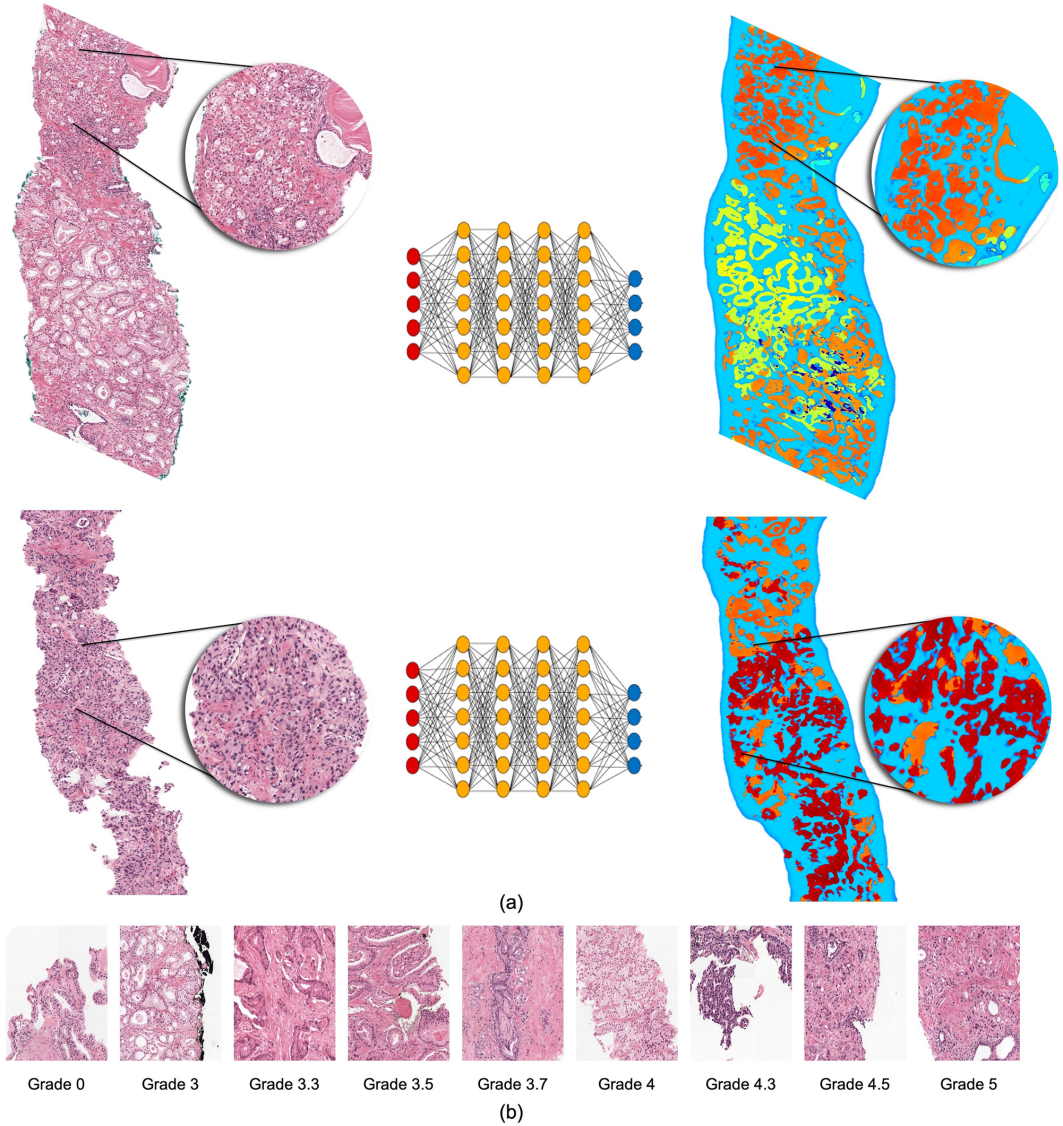
(**a**) Heatmap illustrating the transition in terms of likelihood probabilities of Gleason pattern. (**b**) Continuous Gleason pattern learning by the system. The system can discriminate between well-formed glands and those that are poorly formed and assign a finer categorization.

When discrete classifications are applied to glands that appear on a continuous morphological spectrum—such as the Gleason pattern 3/4 transition between small glands and poorly defined acinar structures, or the Gleason pattern 4/5 transition between fused glands and nests or cords—significant additional variability is introduced. Our method can identify these transitions in terms of likelihood probabilities and assign finer-grained Gleason patterns to glands. This is especially true where pathologists disagree on Gleason pattern classification and the underlying histology is likely to fall between patterns, as shown in Fig. 5. This precise classification brings up new research opportunities for more accurate prostate cancer quantification and risk stratification.

Stamey et al.^28^ introduced a new predictive criterion: percent Gleason grade 4/5, which is the proportion of the tumor occupied by high-grade malignancy, to improve the Gleason grading system. The reliability of percent Gleason grade 4/5 in core biopsies has the same inter-observer variability as Gleason scoring, according to Glaessgen et al.^29^. The association between percent Gleason grade 4/5 as scored by pathologists and by the Deep Learning system was explored in this study. The overall correlation (Pearson's coefficient) for the MPUH and Radboud test sets was r = 0.97 for Gleason grade 4 and r = 0.95 for grade 5 (Appendix 2g), indicating that pathologists and algorithm output were in accord.

**Figure 6:**
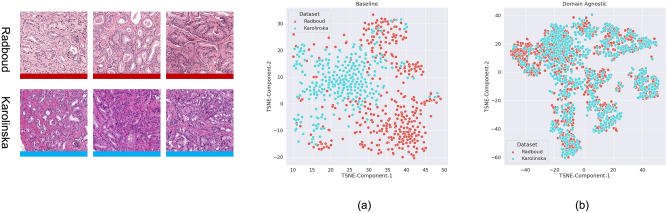
The domain distribution is illustrated. The scatter plots are t-SNE embeddings of 745 picture tiles chosen at random, with representative patches displayed on the left and framed in corresponding colors. The feature map acquired from the activation of the FCN network's last convolutional layer is used to represent each image tile. The results of two experiments are compared: (**a**) representation learned by a baseline FCN model and (**b**) domain agnostic training.

We analyze the output feature vectors from the activation layer of the FCN network using the t-distributed stochastic neighbour embedding (t-SNE) data visualization to evaluate the effect of domain agnostic training methodology (Fig. 6). The scatter plots are t-SNE embeddings of 745 images tiles chosen at random, with representative patches displayed on the left and framed in corresponding colors. The feature map acquired from the activation of the FCN network's last convolutional layer is used to represent each image tile. Each dot in this figure represents a feature vector, and the dots of the same color represent feature vectors of the same data set. As shown in Fig. 6a, for the Radboud and Karolinska datasets, the features emerging from the baseline model (without any domain agnostic training) form discrete clusters in learned representation space. The effect of domain agnostic training is illustrated by the distribution of embeddings in Fig. 6b. The previously fragmented learned representation of the unseen domain now overlaps and displays a smooth distribution. The blue cluster representing the unseen domain is now connected to the rest of the embeddings, increasing the likelihood of improved generalisation results. The prediction performance of the domain agnostic trained model on the test set of images from Karolinska was 83.1 percent (κ_quad_ 0.93). On the other hand, the baseline model, which does not have domain agnostic training, does not generalize well to unseen data, resulting in a 74.6 percent accuracy (κ_quad_ 0.88). Appendix 2h shows the confusion matrix obtained with and without domain agnostic training on the Karolinska test set.

Previous studies of prostate cancer algorithms devoted to analysing histopathology images have indicated lower or similar cancer detection performance characteristics to those found in the current study, as summarised in Appendix 2i. Litjens et al.^7^ developed a Deep Learning CNN with data from 225 slides of prostate cancer needle core biopsies to detect cancer in specimens, with an AUC for the 90th percentile analysis of 0.98. Lucas et al.^14^ found that with sufficient training, the CNN can distinguish areas that are not atypical from malignant with an accuracy of 92%; sensitivity and specificity of 90% and 93% respectively. Arvaniti et al.^12^ used extensive Gleason annotations to train a CNN model. On the test cohort, the inter-annotator agreements (κ_quad_) between the model and each pathologist were 0.75 and 0.71 respectively, equivalent to the inter-pathologist agreement (κ_quad_ 0.71). Nir et al.^15^ evaluated the performance of a classifier for automatic prostate cancer grading by comparing multiple cross-validation procedures. They achieved AUC 0.750 for cancer diagnosis on 230 tissue microarray cores from radical prostatectomy. Campanella et al.^6^ achieved results on the ResNet34 and VGG11-BN of 0.976 and 0.977 AUC respectively for cancer vs. benign classification with 12,160 WSI. Nagpal et al.^9^ used 1557 slides to train a DL system and compared it to a reference standard provided by 29 pathology experts and found that the model had a mean accuracy of 0.61 on the validation set. Raciti et al.^16^ recently published research on how the Paige Prostate Alpha system may affect pathologists when diagnosing prostate cancer. Paige Prostate Alpha enhanced the sensitivity of all pathologists when diagnosing cancer from 74 to 90%. They noted an increase in average sensitivity of 20% for Grade group 1, 13% for Grade group 2, and 11% for Grade group 3, allowing the pathologist to diagnose lower Gleason grade groups better. Bulten et al.^11^ published a large trial in which a deep-learning system outperformed 10 out of 15 pathologists in determining the malignancy of a biopsy (AUC 0.990). Ström et al.^10^ digitized 6682 needle core biopsies slides and 271 external source slides before training a deep learning system on them. The model and designated pathologist had an AUC of 0.960 for identifying cancer and a mean pairwise kappa of 0.62 for assigning Gleason grades. Mun et al.^13^ recently published the YAAGGS deep learning system on a large data set from two hospitals. For Gleason grade group prediction, their technique had a 77.5% accuracy and a kappa of 0.65.

In summary, most previous articles on this subject have focused on algorithms that only predict core-level Gleason grade groups. In contrast, our study reports high-performance characteristics of a multi-task algorithm for prostate cancer interpretation, i.e., malignant vs benign, percentage area of tumor, core-level grading, and percentage area of Gleason scores. Furthermore, most earlier approaches have limited applicability to data distributions that haven't been seen before during training. On unseen test set, our unique domain invariant training mechanism demonstrated better generalizability.

Some limits have an impact on this work, but it serves as inspiration for future development. Misclassifications were occasionally discovered in the output pixel-level overlays, particularly in the stromal regions and at the margins of the tissue borders. The majority of tissue border misclassifications are caused by preparation artifacts that the network does not recognize. An extra neural network might be trained to detect artifact regions and eliminate them as a pre-processing step to avoid such misclassifications in a clinical application. The subjective character of the Gleason scoring system is a more serious issue that is not specific to our study. The current study also found that inter-pathologist heterogeneity in Gleason score annotations is not negligible (mean inter-observer agreement of κ_quad_ = 0.79). Consensus annotations from a large panel of pathologists could help an automated Gleason grading approach. Larger-scale studies involving numerous medical facilities are thus required to consolidate and develop a system that could be used in clinical practice. The Gleason grading methods for biopsies give useful prognostic information. However, no assessment of the algorithm's predictive efficacy or a direct comparison to long-term clinical outcomes was carried out in this investigation. We plan to analyse long-term clinical outcomes from biopsy cases in the future to improve risk stratification.

## Supplementary Information

Supplementary Information.
