# Supplementary material for: A deep learning system for prostate cancer diagnosis and grading in whole slide images of core needle biopsies
Source: Sci Rep. 2022 Mar 1;12:3383. doi: 10.1038/s41598-022-07217-0 (PMC8888647; doi:10.1038/s41598-022-07217-0)
Supplement: Supplementary file 1 — Supplementary Information. [file 41598_2022_7217_MOESM1_ESM.docx]

**Supplementary Material**

**1. Appendix 1**

**a. Implementation**

The multi-task loss function is used to train the combined feature extractor, segmenter, and decoder for image normalization. 512 x 512 patches were created at a magnification level of 40. The model is trained for 200 epochs in mixed-precision mode with a batch size of 16. Cosine Annealing scheduler without warm restarts is used with an initial learning rate of 5e-3. We sample a uniform distribution of images for each label class to avoid class imbalance. Rotation by multiples of 90 degrees, horizontal and vertical flipping were employed as augmentations. All models were trained using the NVIDIA using NVIDIA RTX 6000 GPU, and the code was written in PyTorch.

**b. HED Jitter**

We use HED jitter to generate stain-alternative histopathology images [30, 31]. The method uses colour deconvolution to convert RGB channel images to HED space (Hematoxylin, Eosin, and DAB) in order to identify the contribution of applied stains based on stain specific RGB absorption. The three colour channels are jittered independently in HED space before being transformed back to RGB space. As a result, the samples resemble data from various sources.  The altered image is used to train the stain normalization network, which reconstructs the raw image, as well as to learn stain agnostic features by matching logits of the altered image to the original image. We use a 0.04 intensity value for HED augmentations.

**c. Training parameters**

**
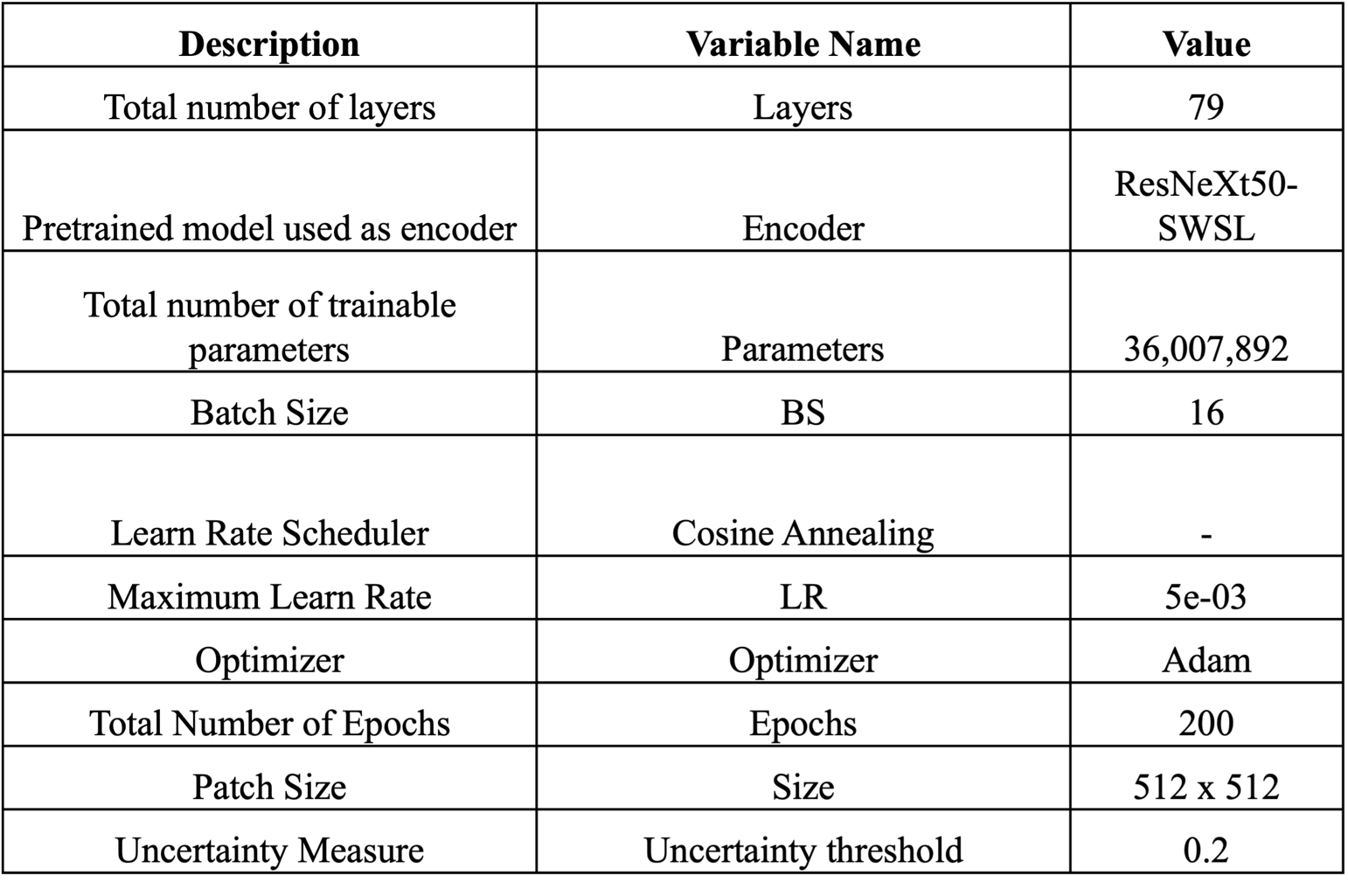
**

*Table I: Training hyperparameters and constants*

**d. Pseudocode**

**
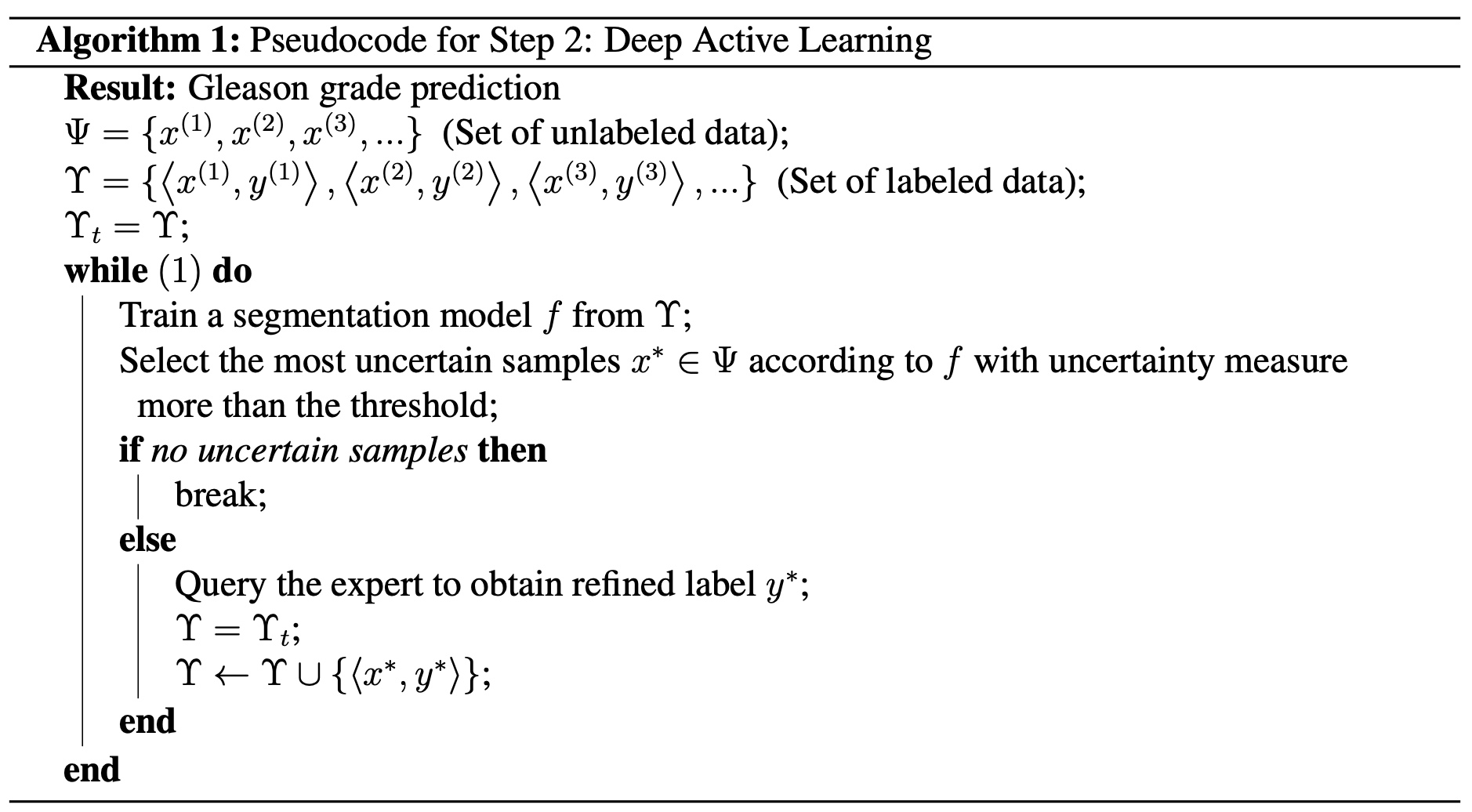
**

**2. Appendix 2**

**a. ISUP grade group distribution**


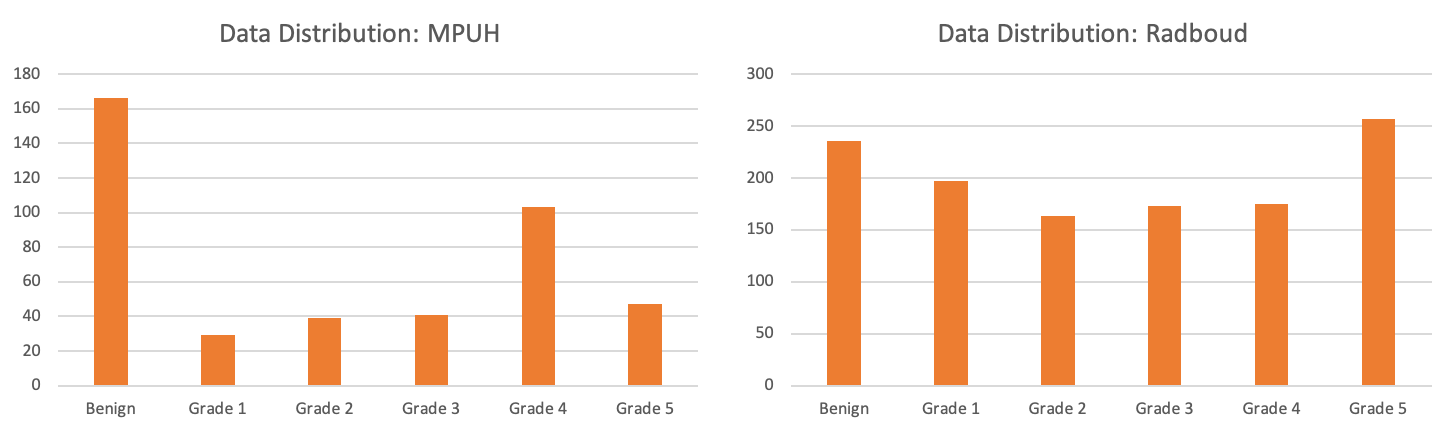


**b. ROC analysis on ISUP grades.
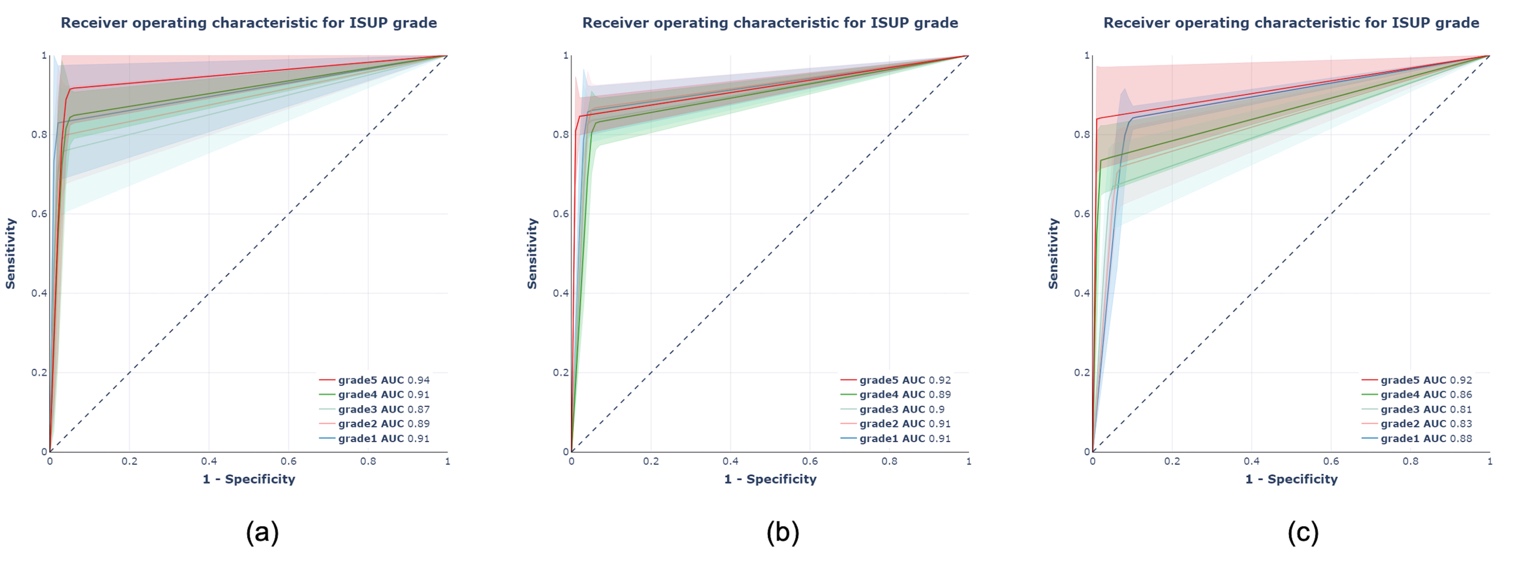
**

*Figure I: ROC analysis on ISUP grades. (a) MPUH; (b) Radboud; (c) Karolinska.*

**c. Stain color variation across different sources**


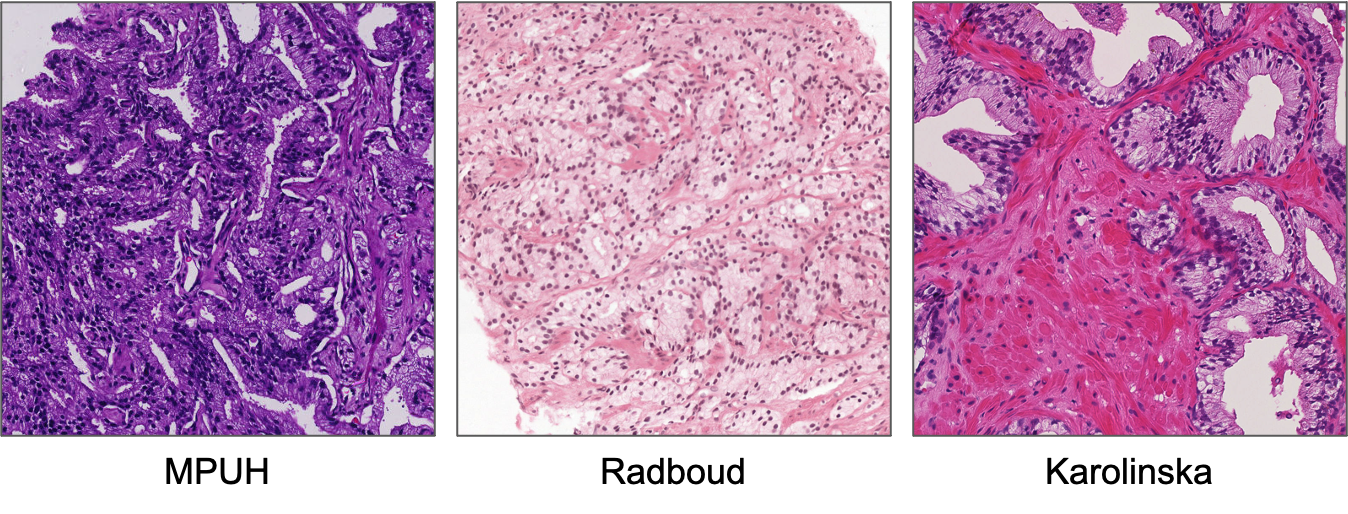


*Figure II: Variation in stain color distribution between biopsies received from three different sources.*

**d. Core-level CNB classification**


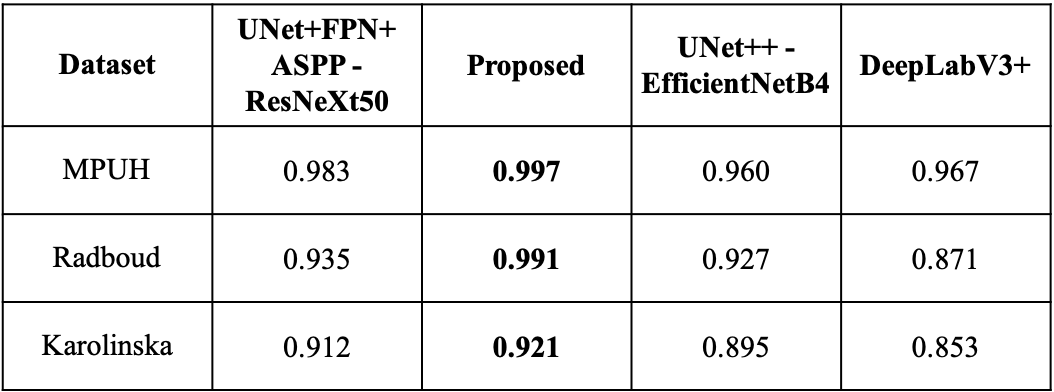


*Table II: AUC analysis for core-level CNB classification task.*

**e. Qualitative results**

*
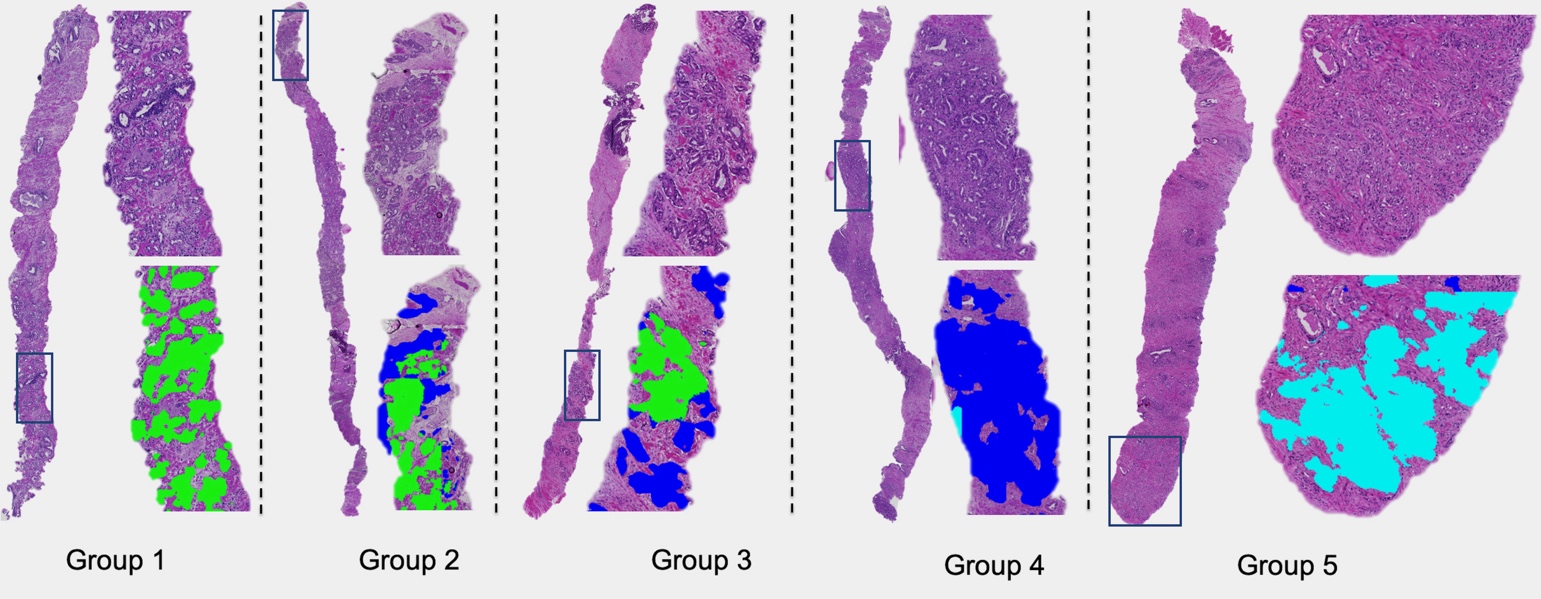
*

*Figure III: Sample examples of positive cases (H&E stain, 10x magnification). Gleason pattern 3 is represented by green, Gleason pattern 4 is shown by blue, and cyan represents Gleason pattern 5.*

**
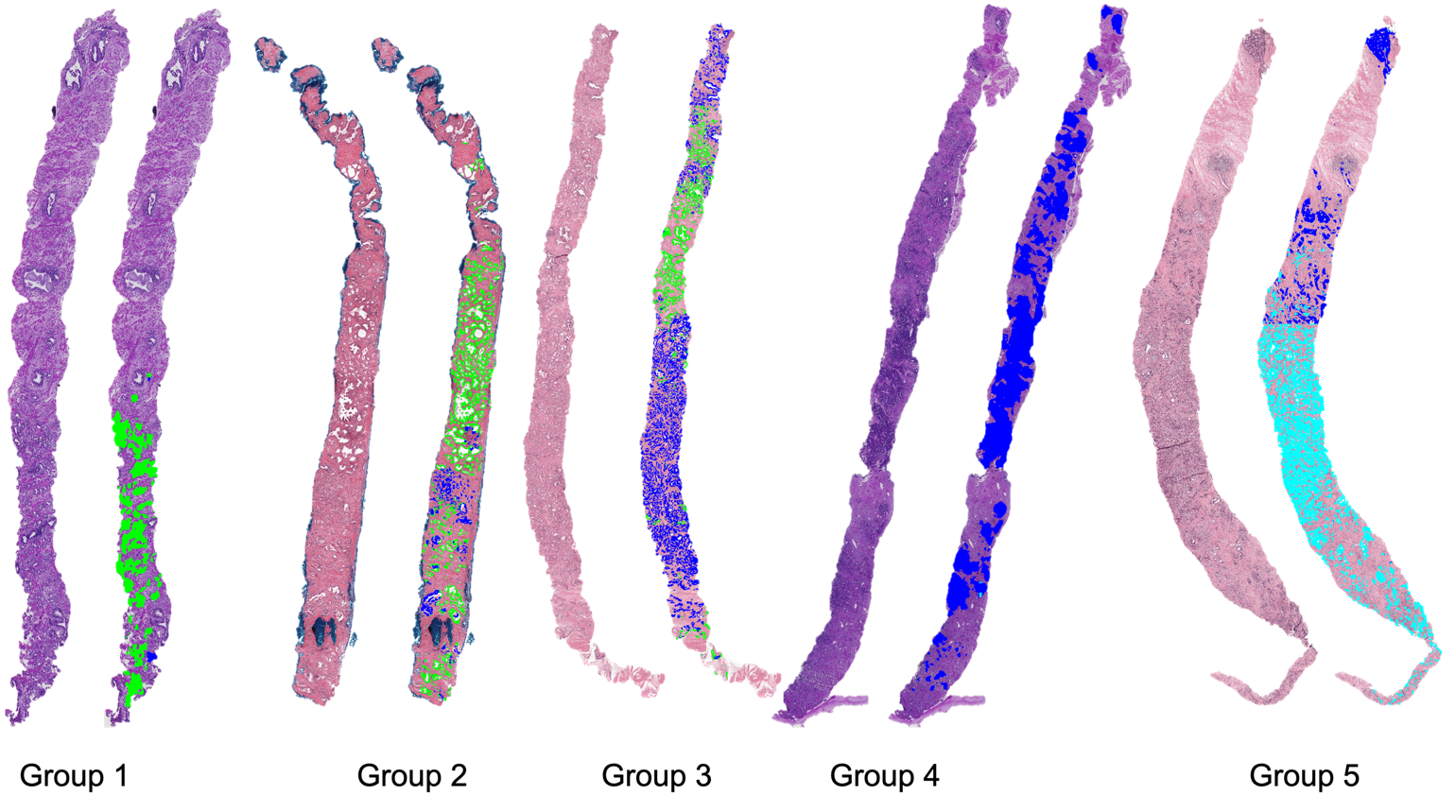
**

*Figure IV: Illustrations of the proposed system's annotations. Gleason pattern 3 is represented by green, Gleason pattern 4 is shown by blue, and cyan represents Gleason pattern 5.*

**f. Example failure cases**


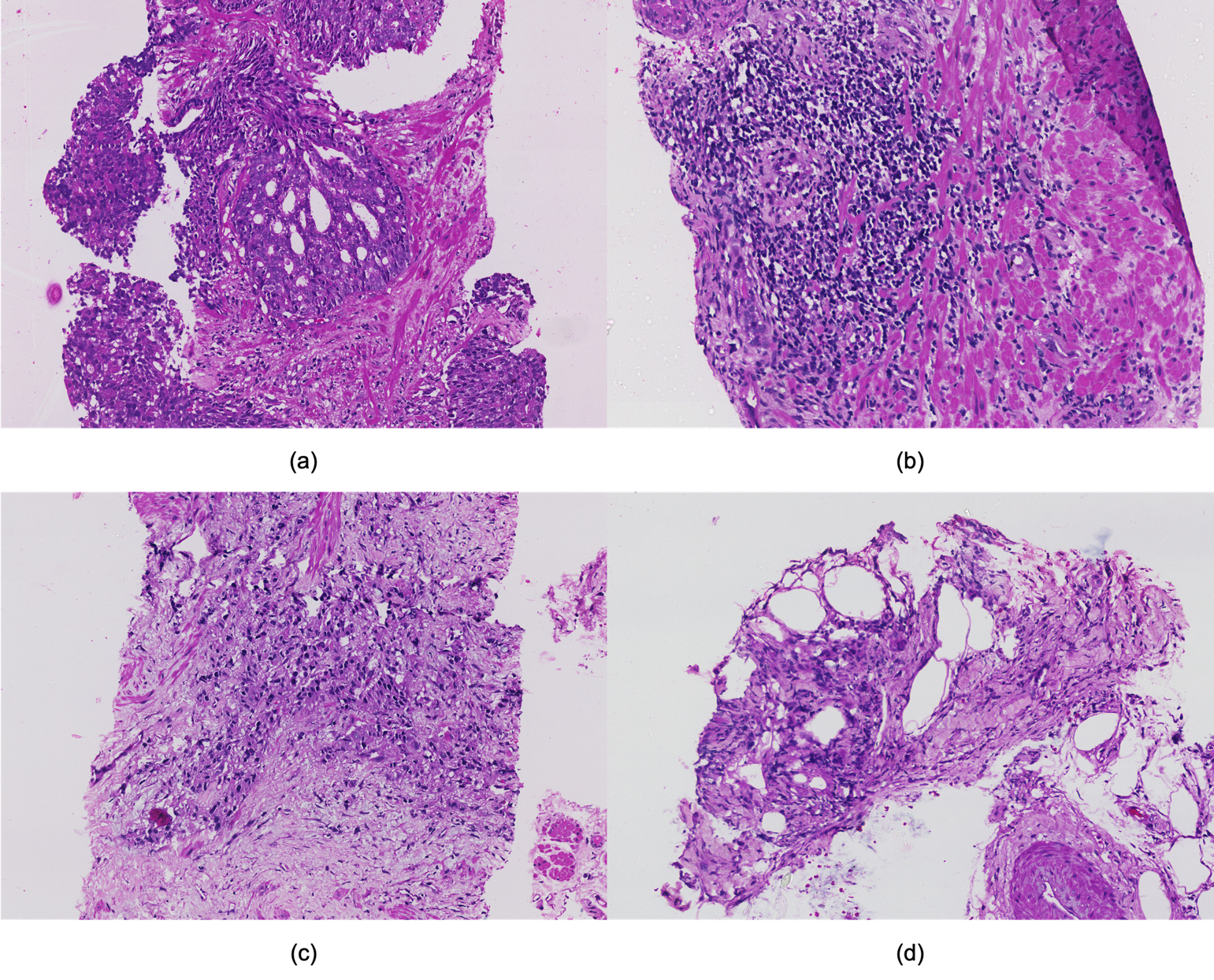


*Figure V: Sample examples of failure cases (H&E stain, 10x magnification).*

**g. Gleason grade 4/5 in core needle biopsies**


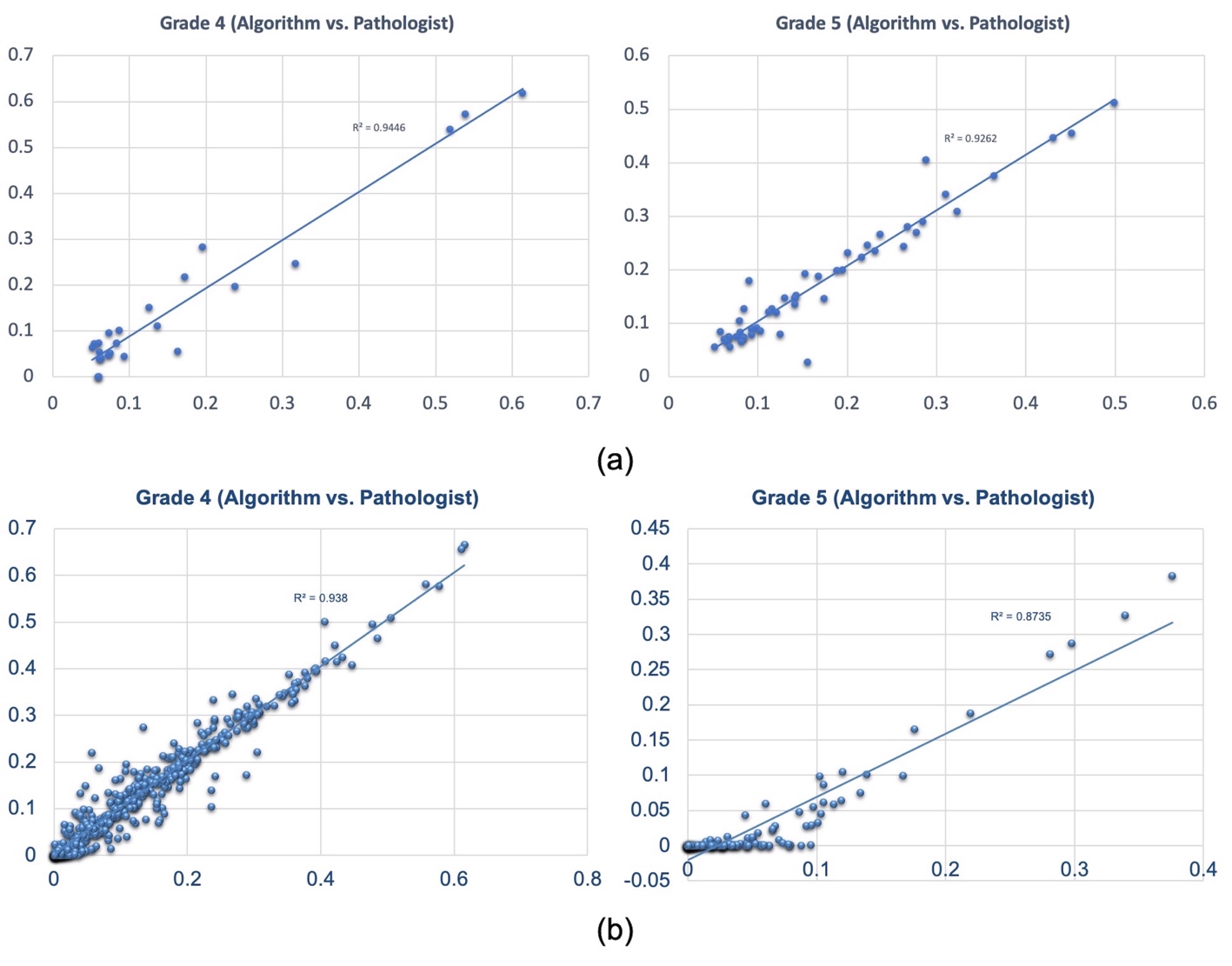


*Figure VI: Scatter plot of percent Gleason grade 4/5 in needle biopsies showing high agreement between algorithm and pathologist annotations.*

**h. Domain agnostic training**


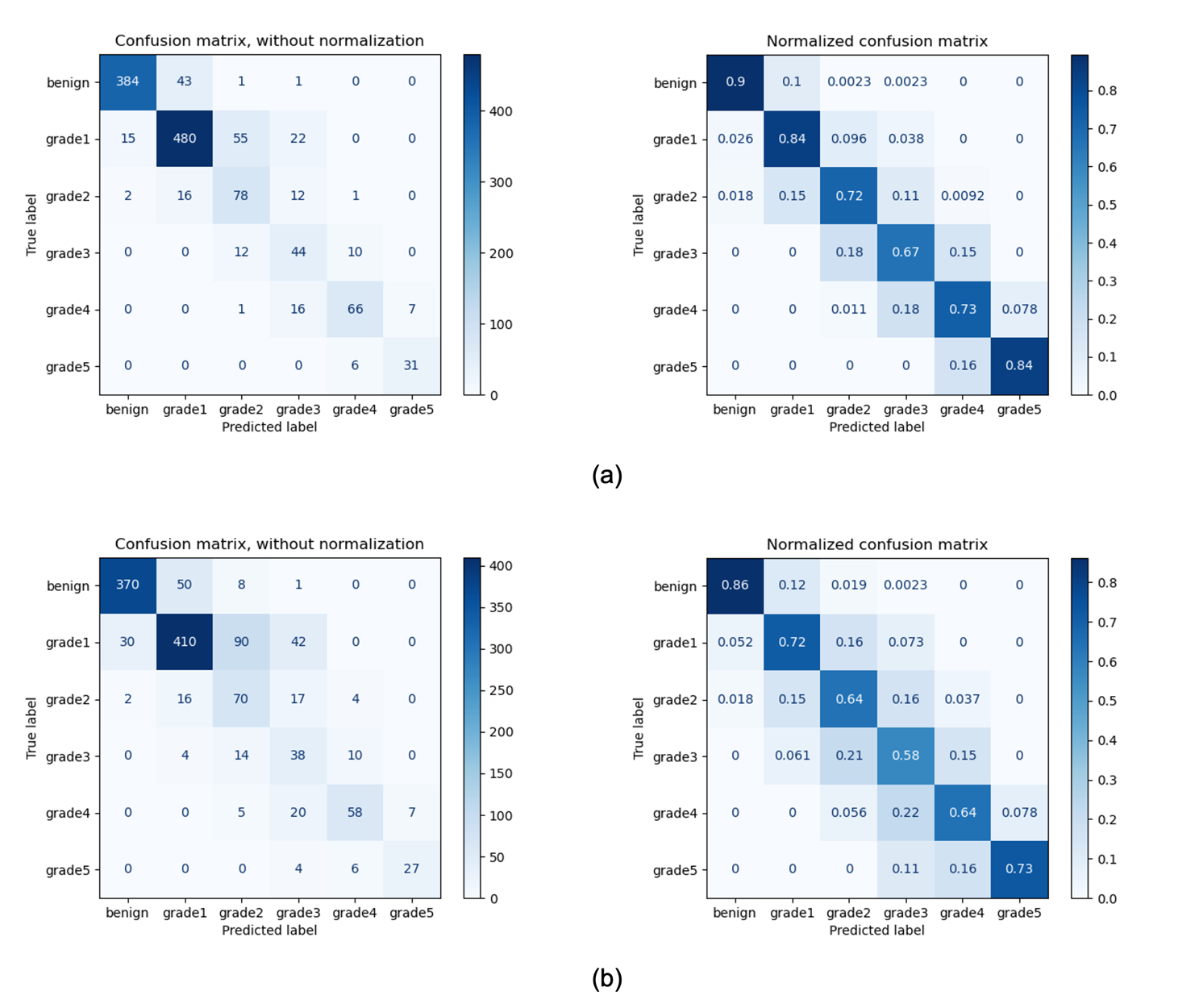


*Figure VII: Confusion matrix depicting the performance of the baseline model (a) with; (b) without domain agnostic training on Karolinska test set.*

**i. Performance of AI based studies**


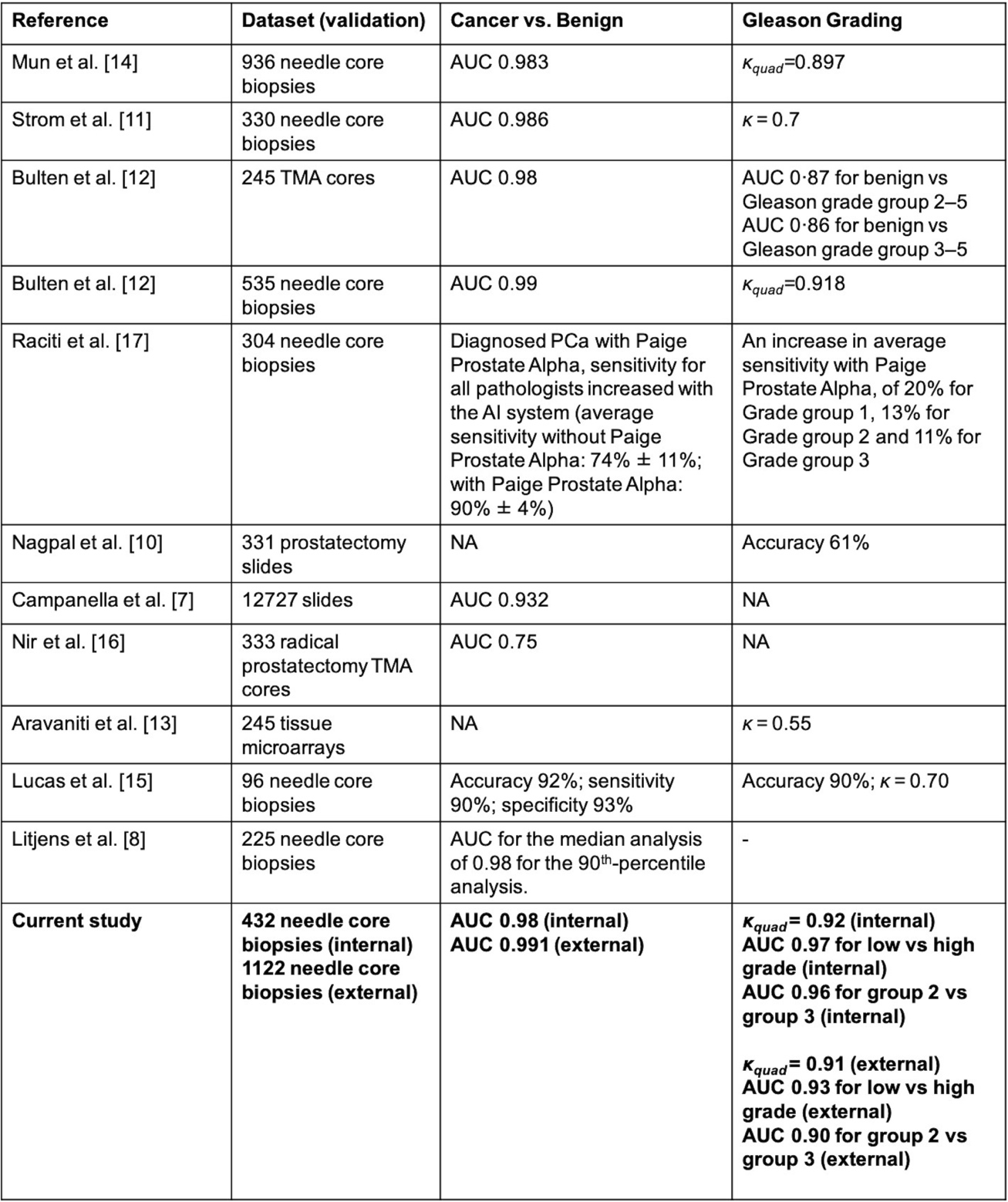


*Table III. Studies describing Performance of AI algorithms studies in detection and grading of*

*prostate cancer.*

**3. References**

[30] David Tellez, Maschenka Balkenhol, Irene Otte-H¨oller, Rob van de Loo, Rob Vogels, Peter Bult, Carla Wauters, Willem Vreuls, Suzanne Mol, Nico Karssemeijer, et al. Whole slide mitosis detection in h&e breast histology using phh3 as a reference to train distilled stain-invariant convolutional networks. *IEEE transactions on medical imaging*, **37(9),** 2126–2136 (2018).

[31] David Tellez, Geert Litjens, P´eter B´andi, Wouter Bulten, John-Melle Bokhorst, Francesco Ciompi, and Jeroen van der Laak. Quantifying the e↵ects of data augmentation and stain color normalization in convolutional neural networks for computational pathology. *Medical image analysis*, **58**, 101544 (2019).
